# Integrative preimplantation genetic testing analysis for a Chinese family with hereditary spherocytosis caused by a novel splicing variant of *SPTB*

**DOI:** 10.3389/fgene.2023.1221853

**Published:** 2023-09-18

**Authors:** Yafei Tian, Yao Wang, Jingmin Yang, Pengfei Gao, Hui Xu, Yiming Wu, Mengru Li, Hongyan Chen, Daru Lu, Hongli Yan

**Affiliations:** ^1^ State Key Laboratory of Genetic Engineering and MOE Engineering Research Center of Gene Technology, School of Life Sciences, Fudan University, Shanghai, China; ^2^ Department of Reproductive Heredity Center, Navy Medical University, Shanghai, China; ^3^ NHC Key Laboratory of Birth Defects and Reproductive Health, Chongqing Population and Family Planning Science and Technology Research Institute, Chongqing, China; ^4^ Institute of Medical Genetics and Genomics, Fudan University, Shanghai, China; ^5^ Shanghai WeHealth BioMedical Technology Co., Ltd. Shanghai, China

**Keywords:** hereditary spherocytosis (HS), haplotyping analysis, single cell whole genome amplification, *SPTB*, preimplantation genetic testing (PGT), noninvasive prenatal testing for monogenic conditions (NIPT-M)

## Abstract

Hereditary spherocytosis (HS), the most common inherited hemolytic anemia disorder, is characterized by osmotically fragile microspherocytic red cells with a reduced surface area on the peripheral blood smear. Pathogenic variants in five erythrocyte membrane structure-related genes *ANK1* (Spherocytosis, type 1; MIM#182900), *SPTB* (Spherocytosis, type 2; MIM#616649), *SPTA1* (Spherocytosis, type 3; MIM#270970), *SLC4A1* (Spherocytosis, type 4; MIM#612653) and *EPB42* (Spherocytosis, type 5; MIM#612690) have been confirmed to be related to HS. There have been many studies on the pathogenic variants and mechanisms of HS, however, studies on how to manage the transmission of HS to the next-generation have not been reported. In this study, we recruited a patient with HS. Targeted next-generation sequencing with a panel of 208 genes related to blood system diseases detected a novel heterozygous variant in the *SPTB*: c.300+2dup in the proband. Sanger sequencing of variant alleles and haplotype linkage analysis of single nucleotide polymorphism (SNP) based on next-generation sequencing were performed simultaneously. Five embryos were identified with one heterozygous and four not carrying the *SPTB* variant. Single-cell amplification and whole genome sequencing showed that three embryos had varying degrees of trisomy mosaicism. One of two normal embryos was transferred to the proband. Ultimately, a healthy boy was born, confirmed by noninvasive prenatal testing for monogenic conditions (NIPT-M) to be disease-free. This confirmed our successful application of PGT in preventing transmission of the pathogenic variant allele in the HS family.

## 1 Introduction

Hereditary spherocytosis (known as well as Minkowski Chauffard disease) is the most common hereditary hemolytic anemia (Da Costa et al., 2013; Wu, Liao, and Lin, 2021). The prevalence of HS is approximately 1/2000 in Europe and North America (Perrotta, Gallagher, and Mohandas, 2008; Bianchi et al., 2012; Da Costa et al., 2013). In mainland China, the prevalence of HS in neonates was indicated to be 1:100,000 (Wang et al., 2015), which may be underestimated due to unbalanced medical resources. The clinical manifestations of HS are highly heterogeneous in terms of the genetic and molecular basis of the disease (Perrotta, Gallagher, and Mohandas, 2008; Xue et al., 2020). The clinical features are pallor due to anemia, jaundice due to hyperbilirubinemia, and splenomegaly. Jaundice is probably the most important sign in neonates (enlargement of the spleen is often absent). In late infancy and adulthood, the typical triad of hemolytic anemia (pale/replicative anemia, jaundice, and splenomegalysis) associated with gallstones is noteworthy (Da Costa et al., 2013). The laboratory diagnosis of HS mainly relies on red blood cell (RBC) morphology examination, osmotic fragility (OF) test, acidized glycerolysis test (AGLT), and maleimide eosin (EMA) (Farias, 2017). Although the EMA test showed maximum disease specificity, no single test can identify all cases of hereditary spherocytosis (Bianchi et al., 2012; Ciepiela et al., 2016). At present, molecular tests based on next-generation sequencing technology are widely used in the differential diagnosis and confirmation of pathogenic variants, further improving the accuracy and specificity of the diagnosis of HS (Xue et al., 2019; Richmond et al., 2020; Xue et al., 2020; Fan et al., 2021; Xie et al., 2021). The diagnosis of HS is the result of a collaboration between the clinician and the laboratory, who should consider family history and rule out other causes of secondary spherocytosis (Farias, 2017; Xue et al., 2019).

Mutations in *ANK1*, *SPTB*, *SPTA1*, *SLC4A1,* and *EPB42* have been associated with HS (Perrotta, Gallagher, and Mohandas, 2008; Da Costa et al., 2013). In terms of the pathogenesis of HS, pathogenic gene variants lead to dysregulation or abnormal erythrocyte membrane proteins, thus reducing the stability and deformability of red blood cells, increasing the permeability vulnerability of red blood cells, and making them easy to rupture and cause hemolysis (He et al., 2018). HS is most commonly associated with autosomal dominant (AD) inheritance, although sporadic and autosomal recessive inheritance have also been reported (Park et al., 2016). 20% of HS cases are caused by mutations in *SPTB*, which encodes the β-spectrin protein (Da Costa et al., 2013). *SPTB* mutations can lead to autosomal dominant inheritance of HS, leading to secondary protein deficiency (Bolton-Maggs et al., 2012). In our previous research, pathogenic variants can be inherited from parents or be *de novo* in the probands, both of which are common (Fan et al., 2021). No homozygous mutation of *SPTB* has been identified to date. In our study, a heterozygous variant in *SPTB*: c.300+2dup and a heterozygous variant in *SPTA1*: c.6631C>T (p.R2211C) were discovered in the proband. According to ACMG guidelines, the variant of *SPTA1* was ultimately rated as a variant of unknown significance (PM2_Supporting + BS4) and the variant of *SPTB* was rated as pathogenic (PVS1+PS2+PM2_Supporting + PP4).

Preimplantation genetic testing (PGT) is a procedure for the genetic analysis of embryos using cells obtained during embryo biopsy. PGT can be used to detect aneuploidies (PGT-A); PGT for monogenic/single gene defects (PGT-M); and PGT for chromosomal structural rearrangements (PGT-SR) (Zegers-Hochschild et al., 2017). PGT-M is an increasingly used technique that allows couples affected by, or known to be at risk for, a genetic disorder to avoid passing it on to the next-generation (Lee et al., 2020). As an issue of reproductive freedom, PGT-M is ethically acceptable (Ethics Committee of the American Society for Reproductive Medicine. Electronic address and Ethics Committee of the American Society for Reproductive, 2018). The use of PGT-M has been reported for hematological disorders such as thalassemia (Kakourou et al., 2018; Satirapod et al., 2019; Chen et al., 2020; Piyamongkol et al., 2022a), and Hemophilia (Bai et al., 2020). However, no research on PGT-M has been reported for the most common HS. In this study, the HS female carrier harbors a splicing pathogenic variant of *SPTB*. The couple has a 50% risk of transmitting the pathogenic variant to the offspring in each pregnancy. After ethical approval, successful PGT procedures, prenatal diagnosis, and careful pregnancy management were performed, and a healthy baby boy was born.

## 2 Materials and methods

### 2.1 Study participants

A 29-year-old woman came to the Department of Reproductive Heredity Center, Changhai Hospital, for genetic counseling to prevent the transmission of HS to the next-generation. She was married for half a year, had a normal sex life, was not pregnant for half a year without contraception, had regular menstruation, a large amount, no dysmenorrhea, and no intercourse pain. The shape of her spleen was enlarged and a splenectomy was done as previous treatment. After obtaining the written informed consent of the couple, we collected the genomic DNA of peripheral venous blood leukocytes and their clinical data. This study followed the principle of the “Helsinki Declaration” and was approved by the Ethics Committee of Changhai Hospital, Navy Medical University (2022-D-1173).

### 2.2 Targeted-sequencing

Genomic DNA was isolated from 2 to 5 mL of subjects’ peripheral blood samples using a commercial kit (TIANGEN, China). The targeted gene capture process was completed in MyGenostics, Beijing, China. Targeted next-generation sequencing with a panel of 208 genes (Supplementary Table S1) related to blood system diseases was adopted to detect the variants. The library was sequenced using a NextSeq500 instrument.

### 2.3 Variant confirmation

Based on the patient’s phenotype, genotype, and genetic pattern, primers were designed for the candidate pathogenic variant sites for confirmation, and at the same time perform Sanger sequencing on the parental candidate sites. The RNA was extracted from the blood of the patient and 1 healthy control, and reverse transcribed into cDNA by RT-PCR. Primers were designed for the target site. Using cDNA as a template, the target site was amplified by PCR and agarose gel electrophoresis, and Sanger and next-generation sequencing were carried out. Forward primer: GCT​GCT​GGA​GGT​GCT​CTC​T; Reverse primer: ACC​ACA​ACA​CAC​ACG​CAT​CC. The variants are described according to the Human Genome Variation Society (HGVS).

### 2.4 Single-cell whole genome amplification

One to three blastocyst trophoblast cells were extracted and dissolved in 4 µL PBS preservation solution. Single Cell amplification was performed using the REPLI-g Single Cell Kit (150343, QIAGEN), following the kit instructions. Specifically, the cell lysis solution Buffer D2 (DTT, 3 µL; Buffer DLB, 33 µL) was prepared first, and 3 µL Buffer D2 was added into 4 µL PBS solution containing single-cell samples for the lysis reaction (65°C, 10 min; 4°C, hold; Hot cover, 70°C), add 3 µL stop solution to the reaction solution after the reaction; Then PCR Master Mix (H_2_O, 9 µL, REPLI-g sc Reaction Buffer, 29 µL, REPLI-g sc DNA Polymerase, 2 µL) was prepared and 40 µL Master Mix was added into the sample. PCR was performed (30°C, 8 h; 65°C, 3 min; 4°C, hold; hot cap, 70°C). The 2 µL amplified product was tested by agarose gel electrophoresis. Part of the product (15 µL) was used for chip detection, and the other part (15 µL) was stored as backup. The remaining 18 µL were rehydrated to 40 µL and purified by 1.8-fold magnetic beads. The eluate volume was 50 µL for the whole gene construction library. The purified products were quantified using Qubit.

### 2.5 Preimplantation genetic haplotyping (PGH)

Firstly, based on the Asian Screening Array (ASA) chip data, using a waste embryo (embryo with a low rating, unsuitable for transfer) as the proband (the lowest allele drop-out rate), the parent target genes and the upstream and downstream haplotypes of a total of 1 Mb were derived. Then, the parental haplotype information combined with the embryo genotype information was used to deduce the embryo haplotype based on the Mendelian inheritance law. Among them, the mother’s haplotype was determined using SNP loci where the mother is heterozygous and the father is homozygous, combined with the genotype of the corresponding loci of the proband, and derived based on the rule of Mendelian inheritance. Similarly, paternal haplotypes require the selection of SNPs that are heterozygous in the father and homozygous in the mother, combined with the genotype of the proband, and derived based on the rule of Mendelian inheritance. Since the target variation in this family is derived from the mother, only the mother haplotype needs to be derived.

### 2.6 Noninvasive prenatal testing for monogenic conditions (NIPT-M)

cfDNA (cell-free DNA) sampling vessel was used to collect 10 mL of the pregnant woman’s venous peripheral blood, and an EDTA sampling vessel was used to collect 5 mL of the father’s venous peripheral blood. gDNA was extracted using the magnetic bead method Blood genomic DNA small amount extraction kit (Shanghai Enlighten Biotechnology Co., LTD., LDB747). The blood sample of the pregnant woman was collected at 1600 g, and centrifuged at 4°C for 10 min, and the supernatant was collected into a new centrifuge tube and continued to centrifuge at 4°C for 1600 g for 10 min. The supernatant was collected for further use. The initial plasma sample size of the pregnant woman was 900 µl cfDNA was extracted using a cell-free DNA extraction kit (Shanghai LifeFeng Biotechnology Co., LTD., DK607), and the final elution volume was 50 µL. All experiments were performed according to the kit instructions. The library was constructed using the Hieff NGS OnePot DNA Library Prep Kit for Illumina kit (Yeasen Biotech Co., Ltd.,12203ES96). Hieff NGS MaxUp II DNA Library Prep Kit for Illumina All-purpose DNA library Construction Kit (Yeasen Biotech Co., Ltd., 12200ES96) was utilized to build the library. QSEP100 (BIOptic, China) was used for fragment analysis and Qubit 4.0 (Thermo, United States) was used for quantitative analysis. Twist Standard Hybridization and Wash Kit (Twist Bioscience, United States) was used to hybridize and capture the constructed whole genome library. The captured library was sequenced by MGISEQ-T7. Finally, the sequencing error rate and fetal cffDNA (Cell-free fetal DNA) concentration were calculated to construct family haplotype and fetal haplotype prediction.

## 3 Results

### 3.1 Clinical description

**FIGURE 1:**
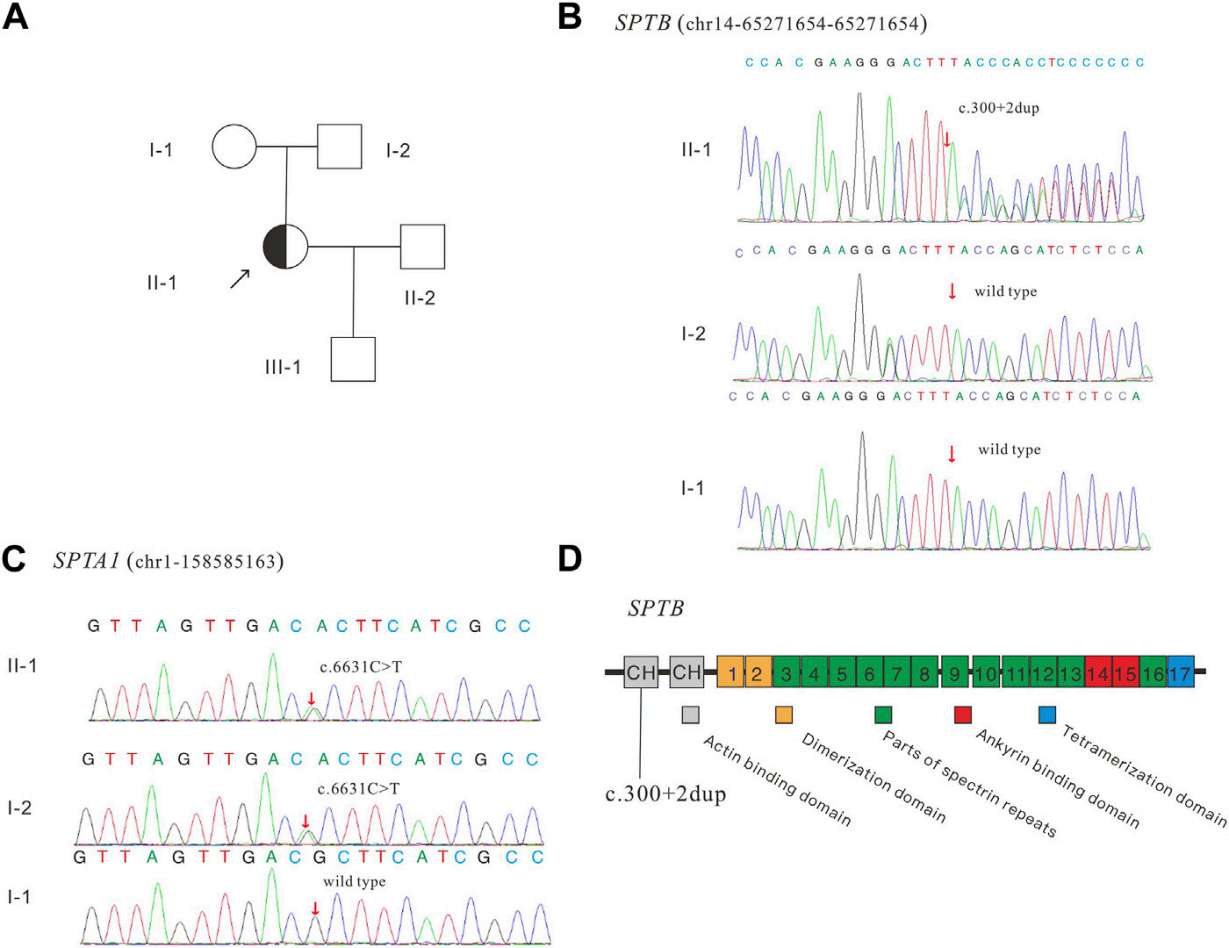
Pedigree of the family **(A)**, Sanger sequencing of the *SPTB* **(B)** and *SPTA1* **(C)** identified variants, and schematic diagram of the β-spectrin domains **(D)**. *SPTB* encodes human erythroid β-spectrin, including two N-terminal calponin-homology (CH) domains and 17 repeat sequences. CH (gray box) represents the actin-binding domain, repeats 1–2 (brown box) mediate beta dimer formation, repeats 3–13 and 16 (green box) are the spectrin repeats, repeats 14–15 (red box) represent the anchor-binding domain, and C-terminal repeats (blue box) represent tetramerization.

The patient was admitted to the Reproductive Medicine Center of Changhai Hospital in July 2018. Clinical tests showed that the patient had more spherical cells beyond the normal range in the blood smear, an enlarged spleen, increased osmotic fragility of red blood cells, hyperbilirubinemia, and anemia (Table 1). Based on clinical evidence, the patient was diagnosed with hereditary spherocytosis. The activities of glucose-6-phosphate isomerase, phosphofructokinase, glucose-6-phosphate dehydrogenase, and pyruvate kinase were normal. The patient’s mother had mild anemia, and the patient’s father had a normal phenotype. For further confirmation, genetic testing was performed, and the panel detection of blood-related diseases showed that *SPTB* had a splicing variant (NM_001024858; chr14:65,271,654–65,271,654; exon2; c.300+2dup). This variant is novel and has not been reported in any public database. Family confirmation and segregation analyses showed that the father of the subject had no variant in this site, and the mother of the subject had no variation in this site, suggesting that this was a spontaneous variant (Figure 1).

**TABLE 1:** Hematological characteristics of the pedigree.

	RBC(T/L)	HCT (L/L)	Hb(g/L)	Total Bilirubin (μmol/L)	Direct Bilirubin (μmol/L)	Indirect Bilirubin (μmol/L)	Ret (Fraction %)	OF(g/L) (start-hemolysis)	OF(g/L) (start-hemolysis)	AGLT_50_
Proband	2.7	0.24	82	71	5.8	65.2	13.79	0.6	0.42	60"
Mother	4.27	0.33	95	NA	NA	NA	0.78	0.48	0.3	>290"
Father	4.43	0.43	120	NA	NA	NA	0.85	0.45	0.3	>290"
References for female	3.5–5.0	0.37–0.47	110–115	5.13–20.53	<5.13	1.71–17.10	0.5–1.5	0.42–0.45	0.27–0.30	>290"
References for male	4–5.5	0.42–0.49	120–160	5.13–20.53	<5.13	1.71–17.10	0.5–1.5	0.42–0.45	0.27–0.30	>290"

HCT, hematocrit; Hb, hemoglobin; Ret, reticulocytosis; NA, not available; OF, osmotic fragility.

### 3.2 Variable splicing validation

**FIGURE 2:**
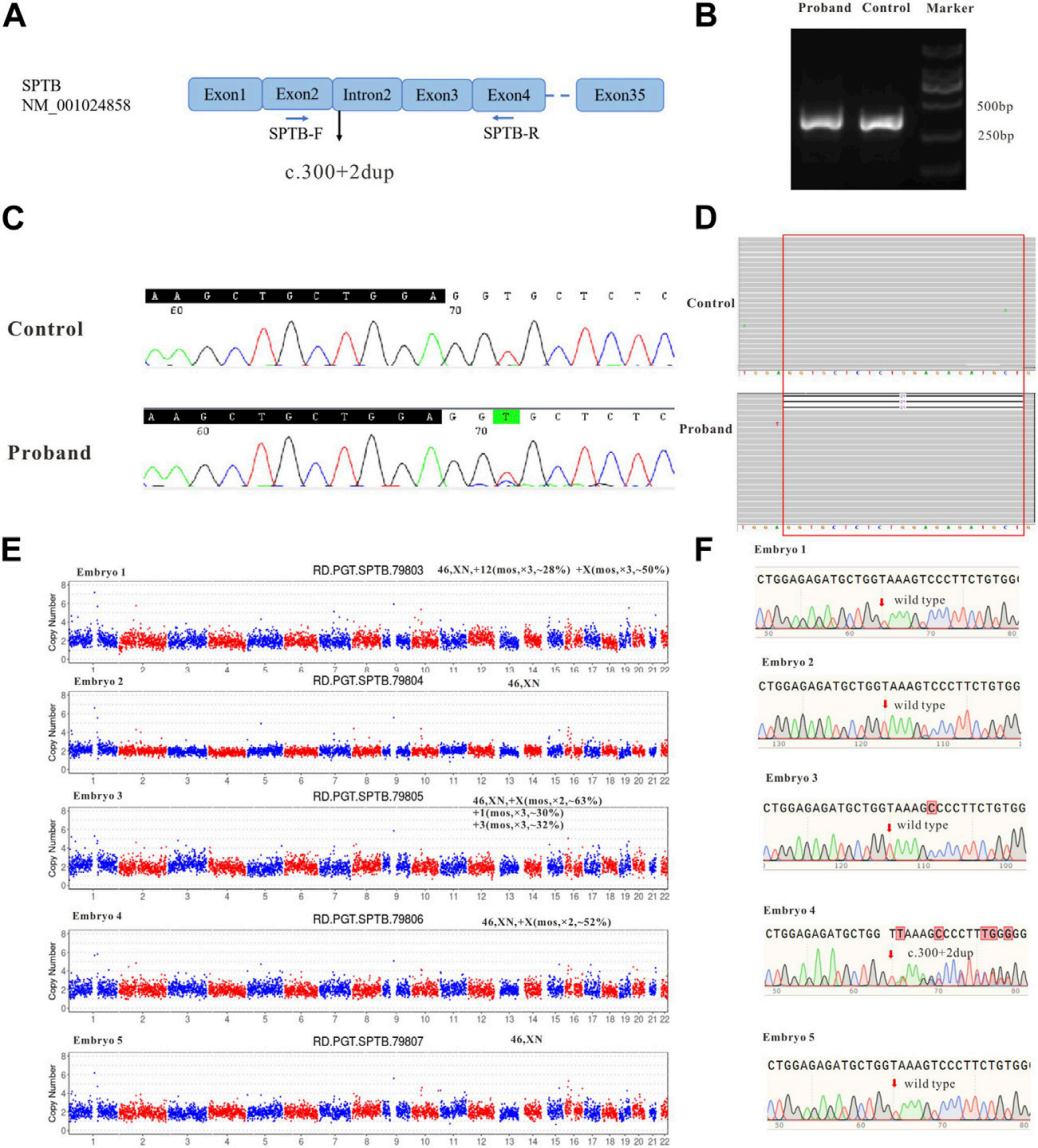
The effect of the identified variant (*SPTB*: NM_001024858: c.300+2dup) on mRNA splicing of *SPTB* and CNV and Sanger sequencing results for embryos. **(A)** represents the schematic diagram of the primers and variant site. **(B)** is the result of electrophoresis of PCR products. **(C)** represents the Sanger sequencing results of PCR products of the proband and control; **(D)** shows the next-generation sequencing results of PCR products. **(E)** shows the CNV results of embryos 1–5. **(F)** shows the Sanger sequencing traces for the region of the *SPTB* variant at each embryo’s target site.

To verify whether the variant (*SPTB*: NM_001024858: c.300+2dup) affects the mRNA splicing of *SPTB*, the peripheral blood RNA of the patient and a healthy control was extracted and inversely transcribed into cDNA by RT-PCR. PCR primers were designed to amplify the CDS region near the variant (Figure 2A). The theoretical size of the amplified fragment was 400 bp. The results of gel electrophoresis showed that the amplified fragment length of the patient was not significantly different from that of the control and theoretical amplification (Figure 2B). The cDNA amplification products of patients and healthy controls were sequenced, and the Sanger sequencing results showed that there were low signal peaks at c.279 (Figure 2C, starting after the black background base), but not in the control samples, indicating that c.300+2dup variation may affect RNA splicing. The cDNA products of the patient and healthy control were sequenced using next-generation Illumina sequencing. These sequencing results showed that 21 bases (missing GGT​GCT​CTC​TGG​AGA​GAT​GCT) were deleted at c. 279–299 (Figure 2D, red frame), while no deletion was found in the control samples, indicating that c.300+2dup variation affects RNA splicing.

### 3.3 CNV analysis and detection of pathogenic variant allele

During the *in vitro* fertilization cycle, 14 MII oocytes were obtained, sperm were injected into the eggs by ICSI (Intracytoplasmic sperm injection), and finally 5 developed into blastocysts. The trophoblast was biopsied by laser on the fifth and sixth day after fertilization. 1-3 cells were extracted from the trophectoderm (TE) of each blastocyst and prepared for WGA. All the samples were amplified and verified by agarose gel electrophoresis. CNV-seq sequencing (CNV-seq) results based on NGS are summarized as shown in Figure 2E. The results showed that only embryo 2 and embryo 5 had normal karyotypes. The chromosome abnormalities of the other three embryos were related to different degrees of chromosome chimerism, especially E03, which showed multiple chromosome chimerism. The carrying status of the *SPTB* variant of each embryo was first verified by Sanger sequencing following the same protocol as for the blood DNA samples. The results of Sanger sequencing showed that none of the other four embryos contained c.300+2dup variant except embryo No. 4.

### 3.4 SNP haplotyping

**FIGURE 3:**
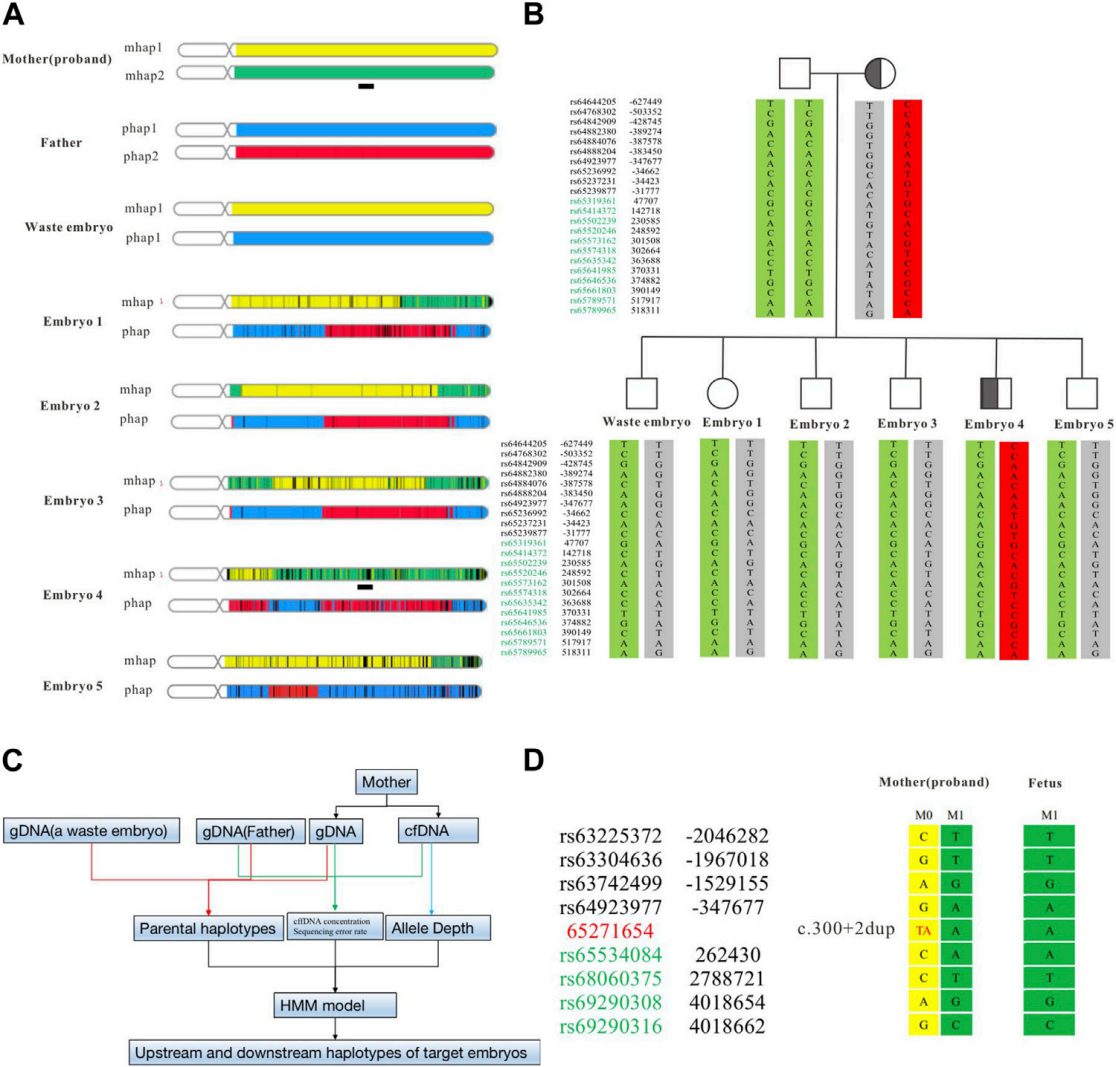
Haplotype construction based on SNP sites of ASA chip and NIPT-M results based on targeted capture. The haplotype results at the overall level of the chromosome constructed by the ASA chip **(A)**. The haplotype of the parent and embryo **(B)**. The analysis flow of NIPT-M **(C)**. The fetal and maternal haplotypes **(D)**. The yellow part is M1 (parent source), the green part is M2 (parent source), the blue part is P1 (parent source), and the red part is P2 (parent source). The black square schematic part [Chr14:63,248,804–67,271,654 (hg19)] is a total of 4 Mb region upstream and downstream of the identified variant in *SPTB*.

Some embryos carrying the *SPTB* variant had been detected by Sanger sequencing, however, the result was based on whole-genome amplification products from embryonic biopsy cells. Allele dropout (ADO) is difficult to avoid, which may lead to misdiagnosis. To reduce its interference with the diagnosis results, we used SNP markers in the 1 Mb region to the side of the target gene for linkage analysis (Figure 3A). The ASA chip we used is a genome-wide SNP chip based on 9000+ East Asian genome sequencing data from Illumina. It contains 700,000 markers with good coverage for low-frequency variants in East Asia (MAF is 1%–5%). In this study, 22 effective SNPs were obtained to construct the mother haplotype. A waste embryo was used as the proband, and a total of 4 Mbp upstream and downstream of the target variant was used for haplotype analysis. According to the results of the ASA chip, we analyzed all SNP loci on parents and embryos and found that all embryos had normal haplotypes except embryo No. Four and the mother carrying the same *SPTB* variant (Figure 3B).

### 3.5 Embryo transplantation and prenatal genetic diagnosis

Regarding the decision to transfer the embryos, the parents had discussions with reproductive doctors, embryologists, and medical geneticists at the Center for Reproductive Medicine at Changhai hospital. According to the results of PGT-M and CNV analysis, the final decision was made to use embryo 2 for transplantation. Four weeks after transplantation, the B-ultrasound showed a fetal heartbeat, indicating a successful pregnancy. At 13 weeks of gestation, maternal peripheral blood was collected for NIPT-M detection according to the procedure shown in Figure 3C, and the results showed that the fetus did not carry the disease-causing variant. The pregnant woman underwent amniocentesis at 20 weeks of gestation for prenatal genetic diagnosis. The prenatal diagnosis (Figure 3D) was consistent with preimplantation genetic testing. The woman eventually gave birth to a healthy boy who did not carry the variant. All blood indicators were normal after birth.

## 4 Discussion

Hereditary spherocytosis is a heterogeneous disease characterized by spherical erythrocytes on a group of peripheral blood smears. The clinical features of these diseases are anemia, jaundice, and splenomegaly, and their severity varies. Common complications include cholelithiasis, hemolytic attacks, and aplastic crisis (Perrotta, Gallagher, and Mohandas, 2008). The laboratory diagnosis of HS mainly relies on red blood cell (RBC) morphology examination, osmotic fragility (OF) test, acidized glycerolysis test (AGLT), and maleimide eosin (EMA) (Bianchi et al., 2012; Bolton-Maggs et al., 2012). Although these methods have high accuracy, none of them can detect all HS patients (Bianchi et al., 2012). With the progress of molecular diagnostics, targeted panel sequencing based on next-generation sequencing has been widely used in rare diseases (Kamps et al., 2017; Svidnicki et al., 2020), which can enrich the target region genes with high depth. It can help us quickly find the cause of a genetic disease and understand the pathogenesis behind it (O'Donohue et al., 2022). As a powerful auxiliary diagnostic tool, genomic diagnostics will play a more and more important role in the examination of rare diseases.

SPH1 (Spherocytosis, type 1), SPH2 (Spherocytosis, type 2), SPH3 (Spherocytosis, type 3), SPH4 (Spherocytosis, type 4), SPH5 (Spherocytosis, type 5) are caused by *ANK1*, *SPTB*, *SPTA1*, *SLC4A1*, and *EPB42* gene mutations, respectively (He et al., 2018). In our case, heterozygous variants were found in both the *SPTB* and *SPTA1* genes. The variant (c.6631C>T, p.R2211C) in the *SPTA1* is classified as a variant of unknown significance (VUS). The heterozygous variant (c.300+2dup) of the *SPTB* gene, which can lead to autosomal dominant inheritance of HS, is classified as pathogenic. Combined with the genotype, phenotype, and genetic pattern of the proband, we finally determined that the variant of *SPTB* was the likely cause of HS.

PGT-M has been in development for more than three decades since Handyside first reported clinical use of preimplantation genetic diagnosis (PGD) for recessive X-chromosome -linked diseases in 1990 (Handyside et al., 1990). However, there may be a risk of misdiagnosis (for example, Allele dropout (ADO)) in the process of PGT. Advanced detection techniques such as linkage analysis have been utilized, making PGT safer and more practical. Linkage haplotype analysis based on SNP is the key to avoiding misdiagnosis caused by inherent defects of ADO or PCR methods (Destouni et al., 2018; Luo et al., 2019; Zhao et al., 2019; Che et al., 2020; Li et al., 2020; Hu et al., 2021; Peng et al., 2021; Malcov et al., 2023). Here, we report a case in which a mother with an *SPTB* pathogenic variant was successfully involved in PGT. In addition, to support the successful application of PGT, we verified that the fetus did not contain the pathogenic variant by NIPT-M and CMA chip technology. Prenatal gene diagnosis is the key to evaluating the true status of pregnancy outcomes. After the newborn was born, we examined him in detail and the results showed that the newborn was in good health.

Currently, PGT offers a crucial alternative strategy to avoid the transmission of pathogenic variants for couples diagnosed with rare monogenic diseases through carrier screening or other genetic diagnoses (Xi et al., 2020; Yang et al., 2022a; Bai et al., 2022; Piyamongkol et al., 2022b; Yang et al., 2022b; Zhang et al., 2022; Xu et al., 2023). In mainland China, as of December 2022, there are 93 institutions that can do PGT, which can basically cover all the Chinese people. However, PGT is still a costly procedure in China, and multi-cycle failures in particular can put a huge burden on some families. Some cities or provinces in China, such as Beijing and Liaoning Province, have included assisted reproduction in the coverage of medical insurance. This will greatly reduce the consumption of patients. The reduction in the cost of technologies such as next-generation sequencing will also promote the continued development of the assisted reproduction industry. With the support of government policies and the reduction of technology costs, it is believed that PGT will benefit more families in low- and middle-income countries (LMICs) in the future.

## 5 Conclusion

In summary, we identified a novel splicing variant in one Chinese HS family through comprehensive genetic analysis, thus enriching the *SPTB* variant spectrum. The variant affects a low percentage of transcripts with abnormalities that may be easily overlooked in routine testing. Our study provides important data for practitioners of genetic disease testing. The successful implementation of PGT-M and NIPT-M prevented the transmission of the *SPTB* pathogenic variant in the HS family, which can serve as an important reference for other clinicians and specialists. In addition, this study also summarized the clinical symptoms of the patient with HS and analyzed the correlation between genotype and clinical phenotype, which is helpful for family members to understand the range of HS-related symptoms and may contribute to further elucidating the pathogenesis of HS.

## Data Availability

The datasets generated for this study can be found in the SRA and GEO database with the following link: https://www.ncbi.nlm.nih.gov/sra/PRJNA1012517, https://www.ncbi.nlm.nih.gov/sra/?term=PRJNA1012843 and https://www.ncbi.nlm.nih.gov/geo/query/acc.cgi?acc=GSE242733.
